# Syndromology at the interface of evolving phenotypes, epimutations, and model systems

**DOI:** 10.1515/medgen-2024-2024

**Published:** 2024-06-06

**Authors:** Nuria C. Bramswig, Dagmar Wieczorek

**Affiliations:** Heinrich-Heine-University Dusseldorf Institute of Human Genetics, Medical Faculty and University Hospital Dusseldorf Moorenstr. 5 40225 Dusseldorf Germany; Heinrich-Heine-University Dusseldorf Institute of Human Genetics Moorenstr. 5 40225 Duesseldorf Germany

The field of syndromology spans a vast range of different and exciting research areas. Many famous clinical geneticists have laid the groundwork for today’s landscape of syndromology. They described the clinical landscape of rare syndromes and started to unravel the underlying genetic causes. The advent of next generation sequencing technologies has led to the discovery of many novel disease-associated genes. This enabled clinical geneticists to increase the diagnostic yield and enlarge patient cohorts, broaden the phenotypic and genotypic spectrum of rare diseases and dig deeper into the underlying pathogenetic mechanisms. In this issue, we want to give an overview of this impressive development of syndromology by assembling a potpourri of different lines of research. We focus on the evolving phenotypes of rare diseases and their clinical management in adulthood, the booming field of epimutations, and the most commonly used model systems for exploration of pathogenetic mechanisms.

Some research groups have started to focus on describing the evolving phenotypes of rare genetic diseases into adulthood. The article by Ariane Schmetz and colleagues gives an overview of these adult phenotypes and emphasizes the need of further studies to improve counselling of families with young children and adults with rare syndromes. They describe the phenotypes of four adult individuals with Costello syndrome illustrating the high variability of neurocognitive outcome and documenting recurrent carcinomata in some individuals.

Dorothee Maliszewski-Makowka and Dagmar Wieczorek describe the developments in and missions of the so-called “MZEB” („Medizinische Zentren für Erwachsene mit Behinderungen“), which were founded to ensure that adults with multiple disabilities receive medical care in an interdisciplinary and multi-professional setting. They summarize the efforts of the MZEB in the LVR clinic in Bedburg-Hau and the Institute of Human Genetics of the Heinrich-Heine University Düsseldorf to employ genetic testing in adults with mental disabilities and developmental and psychiatric disorders.

The article written by Bernhard Horsthemke addresses the growing role of epigenetics in rare diseases by illustrating the classification of epimutations. Despite the large number of disease-associated chromatin modifier genes, for which he gives a comprehensive overview, the pathogenetic mechanisms remain to be elucidated. He concludes that the disease-relevant loci affected by the mutated chromatin modifier genes and their pathogenetic mechanisms are unknown and gives a critical outlook on the clinical application of newly developed epigenome editing tools.

Different model systems are needed to gain insight into these and other pathogenetic mechanisms leading to syndromic diseases, to validate novel disease genes and variants of unknown significance and to assess potential therapeutic means for these rare diseases in a pre-clinical setting. The article by Anne Gregor and Christiane Zweier compiles the most commonly used models and outlines their application areas, as well as their advantages and limitations.

We want to thank all authors for their valuable contributions and hope that the selected topics provide enticing insights into the recent developments in syndromology.

